# A study on habits of tobacco use among medical and non-medical students of Kolkata

**DOI:** 10.4103/0970-2113.76293

**Published:** 2011

**Authors:** T. Chatterjee, D. Haldar, S. Mallik, G. N. Sarkar, S. Das, S. K. Lahiri

**Affiliations:** *Department of Community Medicine and Paediatrics, BMC, Burdan, India*; 1 *Department of Community Medicine, RGKMC, Kolkata, India*; 2 *MMC, Medinipur, Kolkata, India*

**Keywords:** Medical and non-medical students, smokeless tobacco, smoking, tobacco use

## Abstract

**Background:**

Age-old practice “using tobacco” is a well known major global concern as it victimizes all its lovers by a host of chronic noncommunicable diseases including cancer; all develop very slowly and silently, and can cause premature death.

**Objectives:**

To assess the pattern of tobacco use among the medical and nonmedical college students.

**Materials and Methods:**

A cross-sectional descriptive study was carried out in Kolkata collecting anonymous data from 515 medical and 349 nonmedical college students of two medical and two general colleges, selected randomly.

**Result:**

Overall prevalence of tobacco use (18.3% vs 43.6%) and smoking (14.9% vs 40.7%) were significantly less in medical subjects, both across the sex and years of study. Lower rate of tobacco adoption at college level, higher quitting rate, correct knowledge regarding uselessness of filter attached with cigarette, and ill-effects of tobacco consumption were observed among medical participants. More nonmedical subjects were increasingly smoking compared to medical students. Filter-tipped cigarette was the top choice, and smoking was more prevalent mode of use among the nonmedical participants, most (62.3%) of whom were mild users. Curiosity was the top influencing factor for the initiation of tobacco use and two-third users wanted to quit.

**Conclusion:**

Although the mortal habits was comparatively less among medical students, the medical environment seemed to fail to curb the dreadful practice totally. Thereby it can be recommended that active behavior-changing communication is required for all sections of the society to tear out the social root of the problem instead of unimpressive vague health warnings in vogue.

## INTRODUCTION

Tobacco is a serious threat to health[1] and a proven killer and[2] ranks second as a cause of death[3] in the world taking its toll by killing some 5 million people globally. Tobacco use is an emerging pandemic marching forward relentlessly.[4,5] Evidences accumulating since early 1950s indicate that more than 25 diseases are now known or strongly suspected to be causally related to smoking.[6,7] WHO estimates that unless current smoking pattern is reversed, tobacco will be responsible for 10 million deaths per year, by the decade 2020–2030, with 70% of them occurring in developing countries. In India tobacco kills 8–10 lakhs people each year and many of these deaths will occur in people who are very young. It has been estimated that an average of five-and-a-half minutes of life is lost for each cigarette smoked.[6,7] Deaths attributable to tobacco are expected to rise from 1.4% of all deaths in 1990 to 13.3% in 2020. India, as per WHO projection, will have the highest rate of rise in tobacco-related deaths during this period compared to all other countries/regions.[6–8] Youth in general and adolescents in particular fall prey to this deadly habit with severe physical, psychological, and economic implications.[9] Among the youth, students are particularly involved due to increasing academic pressures and uncertain career.[10] Encouragement from peer group, the lure of popularity, and easy availability of tobacco in different forms make a teenager an easy prey.[10] In India, approximately 5500 children and adolescents start using tobacco products daily, some as young as 10 years. The majority of users have first used tobacco prior to the age of 18 years.[11] Teaching about the effects of use of tobacco is essential for college students, both medical and nonmedical, because these would be physicians, future teachers, and other responsible citizens and will hold, specially the physicians, key positions to lead tobacco cessation programs in our community.[12] So they should not be sanctimonious. As the medical students develop and study in the same sociocultural environment during early adolescence period with their present nonmedical peers, their behavior regarding the use of tobacco is expected to be akin, to some extent, to that of their nonmedical peers, at least at the beginning. At college level, gathering of in-depth knowledge and witness of burden of tobacco-related diseases and exposure to more stringent anti-tobacco environment may induce, over the course of time, some form of behavioral change in respect of tobacco use among medical students. On this background, paucity of information regarding the present trend of tobacco use among the medical students of Kolkata, in comparison to their nonmedical counterpart, inspired the investigators to carry out the present work with the following objectives:

1. To assess the magnitude of tobacco use among the medical and nonmedical students
2. To explore the correlation of initiation and continuation of tobacco use
3. To assess their knowledge about ill-effects of tobacco
4. To grasp their opinion regarding tobacco cessation

## MATERIALS AND METHODS

A cross-sectional descriptive study was carried out from 1 August, 2007 to 20 December, 2007 (by August the first-year student would start their classes with good attendance and after Christmas the attendance would be poor because of the examination) in Kolkata involving students of two medical colleges namely Calcutta Medical College, Kolkata; Nilratan Sirkar Medical College, Kolkata, and two nonmedical colleges namely Presidency College, Kolkata, and Surendranath College, Kolkata. For the purpose of selection, the list of general colleges was sought from the University of Calcutta. From the list of medical and general coeducation colleges, the above mentioned medical and nonmedical colleges were selected through simple random technique by lottery. With a view to make two groups to some extent homogenous, only the students of science stream (as they shared almost same sociocultural environment up to 12th standard) of the selected two general colleges were purposively involved in the study. Based on different previous study results[13,14] and assuming a prevalence of 45%, a sample size was calculated as per the formula 4*pq*/*1*^2^ (where *P* being the prevalence, *q* = 1 – *P* and *1* = allowable error around the prevalence). Considering 10% error at 95% confidence interval, the required sample size was to be a minimum of 488.9, that is, approximately 500. With 20% nonresponse rate, it was estimated to be 600. No sampling technique was adopted and all students present in the class were approached to get involved in the study. After seeking administrative approval and verbal consent of the participants, anonymous data were collected from the students (of 1^st^ year to 5^th^ year) present in classes by using a predesigned and pretested self-administered questionnaire prepared in English. As per the suggestion of teachers of selected colleges, it was decided that the students, both medical and nonmedical, of each year would be approached in class, in the presence of their class teacher, in first half of the college session (for better attendance). The purpose of the study was briefed, confidentiality of information was ensured, and purely voluntary nature of their participation was explained to the students before actual data collection. The data were collected regarding age, sex, class/year of initiation of tobacco use, influencing/precipitating factor behind initiation, form of product used, frequency and pattern/mode of use, factors influencing current use, their attitude toward future use, and their knowledge about the safety of filter-tipped cigarette and tobacco-related health hazards. Thus, a total of 522 medical and 363 nonmedical students, i.e., on the whole 885 students were included in the study. “Ever exposed” was defined as having used tobacco at any stage in their life. Those who used tobacco at least once in the last 7 days were called “current user”. For this study, mild, moderate, and heavy users were defined as those used tobacco less than 5, 5–20, and more than 20 times a day, respectively. The data collected were compiled and analyzed using simple proportion and *Z* test.

## RESULTS AND DISCUSSION

Out of the 923 students approached, 885 participated (participation rate=95.9%). After rejecting nonspecific and incomplete responses, finally, responses of 515 medical and 349 nonmedical subjects, i.e., a total of 864 were analyzed. Most of the participants (96.3%) were in the age group 19–24 years, 60% (515) belonged to the medical category, 65.6% to male with almost equal proportion of lady participants in both groups. 17.9%, 21.7%, 25.8%, 27.8%, 6.9% medical and 35.5%, 30.4%, 15.2%, 9.2%, and 9.7% nonmedical participants belonged to 1^st^, 2^nd^, 3^rd^, 4^th^, and 5^th^ years of study, respectively [Table 1].

**Table 1:** Distribution of participants by their years of study, sex, and current tobacco use [*N*=864]

Year of study	Sex	Medical				Nonmedical				*Z, P* Value
		Student	Using tobacco	Prevalence (%)	*Z, P* value	Student	Using tobacco	Prevalence (%)	*Z, P* value	
1^st^ year	M	57	19	33.3	−	79	42	53.2	−	**
	F	35	1	3.9	−	45	4	8.9	−	−
	T	92	20	21.7	*	124	46	37.1	*	2.5, 0.0124
2^nd^ year	M	73	23	31.5	−	73	37	50.6	−	−
	F	39	0	0	−	33	4	12.1	−	−
	T	112	23	20.5	0.21, 0.836	106	41	38.7	0.25, 0.8026	2.9, 0.0038
3^rd^ year	M	89	21	23.6	−	33	28	84.8	−	−
	F	43	2	4.7	−	20	2	10.0	−	−
	T	132	23	17.4	0.79, 0.4296	53	30	56.6	2.4, 0.0164	5.2, <0.002
4^th^ year	M	95	23	24.2	−	19	15	78.9	−	−
	F	48	2	4.2	−	13	1	7.7	−	−
	T	143	25	17.4	0.74, 0.4594	32	16	50.0	1.3, 0.1936	3.5, <0.002
5^th^ tear	M	27	3	11.1	−	23	17	73.9	−	−
	F	9	0	0	−	11	1	9.1	−	−
	T	36	3	8.3	2.12, 0.034	34	18	52.9	1.7, 0.0892	4.6, <0.002
Total	M	340	89	26.2	−	227	139	61.2	−	8.9, <0.002
	F	175	5	2.9	−	122	12	9.8	−	2.4, 0.0164
	T	515	94	18.3	−	349	151	43.3	−	7.9,<0.002

M, male; F, female; T, total;

* *Z* test across the rows.

** *Z* test across the columns

On the whole, 28.5% (95% CI: of 25.6–31.44) of study subjects reported to have tobacco use at present with a prevalence rate of 18.3% (95% CI: 14.97–21.63) and 43.3% (95% CI: 38.4–48.8%) among the medical and nonmedical groups, respectively, showing a significant difference (*Z*=7.9, *P*<0.002, Table 1) in between.

Sex difference in tobacco prevalence was very conspicuous: 40.4% (95% CI: 27.7–53.1) versus 5.7% (95% CI: 3.1–8.4) overall, 26.2% (95% CI: 21.5–30.9) versus 2.9% (95% CI: 0.4–5.5) for medical and 61.2% (95% CI: 55.4–68.0) versus 9.8% (95% CI: 4.5–15.1) for nonmedical participants, respectively, and statistically significant (*Z*=13.1, *P*<0.002; *Z*=8.6, *P*<0.002 and *Z*=12.4, *P* <0.002, respectively) [Table 1]. As per the report of “The India, Global Health Professional Student’s Survey(GHPSS), 2006,” Ministry of Health and Family Welfare (MHFW), Government of India (GOI); female medical students were significantly less than male medical students to have ever smoked cigarette or used other tobacco products.[15] Roy *et al*. in their study “Smoking and drug-abuse among the newly admitted students of medical colleges in West Bengal” in 1981 observed none of the females, at the time of entry in medical college reported current or past use of cigarettes or habit-forming drugs.[16]

Overall tobacco prevalence was significantly higher among nonmedical male subjects with a prevalence of 61.2% in comparison to 26.2% among their medical counterpart (*Z*=8.87, *P*<0.002), and the fact was also true for nonmedical female candidates having a prevalence rate of 9.8% versus 2.9% (*Z*=2.32, *P*<0.0102, (Tables 1 and 2). Further analysis showed that overall and gender-wise prevalence of “smoking only” was significantly low, as well, among the medical group of respondents [Table 2].

**Table 2:** Distribution of tobacco parameters observed in both group of participants

Parameter			Medical (*N*_1_ = 515)	Nonmedical (*N*_2_ = 349)	*Z, P* values
Tobacco prevalence (%)	Sex wise	Male	26.2	61.2	8.9, <0.002
		Female	2.9	9.8	2.4, 0.0164
		Total	18.3	43.3	7.9, <0.002
	Mode wise	Smoking	14.9	40.7	8.3, <0.002
		Chewing	7.4	6.6	1.76, 0.0784
		Both	4.1	4.0	0.07, 0.9442
Exposure rate (%) at college			10.4	31.5	6.0, <0.002
Given up* (%)			16.1	7.9	2.02, 0.0394
Gradual increase in daily dose (%)			25.5	40.8	2.6, 0.0094
Increase in daily use during mental tension (%)			71.3	53.3	2.89, 0.0038
Correct belief regarding filter-tipped cigarette (%)			61.6	41.8	5.8, <0.002
Awareness regarding the ill-effects of tobacco use (%)	Hypertension		84.3	30.4	18.4, <0.002
	C.H.D.		65.1	37.6	8.2, <0.002
	Stroke		48.4	36.3	3.6, <0.002,
	Bronchitis		54.2	44.7	2.75, <0.006,
	Gastritis		53.4	30.7	6.9, <0.002
	Ca-oropharynx		80.2	71.8	2.9, <0.0038
	Ca-esophagus		66.2	32.7	10.3, <0.002
	Ca-stomach		42.7	27.5	4.7, <0.002
	Ca-kidney		22.1	9.46	5.2, <0.002
	Ca-lung		87.6	79.9	2.96, <0.003
	Ca-larynx		85.2	62.5	7.5, <0.002
	Ca-any part of body		14.2	10.3	1.8, 0.0718
Positive attitude toward quitting (%)			67	66.9	0.02, 0.984
Proportional tobacco use (as %)	Smoking		81.9	94.0	3.75, <0.002
	Chewing		40.4	15.2	4.3, <0.002
	Both		22.3	9.3	2.7, 0.007

* % of total (ever-exposed and current) users

The prevalence rate among medical students, as per present study, was less or comparable to the rates found by similar other studies.[13,17–19]

Group-by-group analysis revealed that overall tobacco prevalence was significantly higher among nonmedical participants in each year of study [Table 1] and it was, in respect of first year, going up gradually over the years and increased significantly in the 3^rd^ year of study (Z=2.4, P=0.0164); on the other hand, the prevalence was decreasing gradually over the years among medical students and declined significantly in the 5 ^th^ year of study (*Z* = 2.12, *P*=0.034). This is contrary to the observation that smoking and other drug use increased with time spent in medical college, made by Roy *et al*.[16] Venkataraman *et al*. in a study based on mailed-in questionnaire to 10 batches of students, selected randomly, between the years 1955 and 1988 in a medical college in South India, revealed the prevalence of 39.51% and the trends of smoking appeared to be in three phases: an initial phase comprising of a steady rise to a peak in the late sixties and seventies, followed by a plateau of the prevalence in next 10 years, and ultimately a sharp fall in the last 5 years.[19] Analysis revealed tobacco prevalence to be significantly higher among nonmedical category only in respect of their use in the form of “smoking only,” chewing and combined use were almost equal in both the groups [Table 2].

Further analysis showed that total 65 and 79 medical and nonmedical students, respectively, were exposed to tobacco in their school days, whereas 47 medical and 85 nonmedical participants adopted tobacco at their college life. Thereby, the exposure rates at college were 10.4% and 31.5%, respectively, significantly higher in nonmedical respondents (*Z*=6, *P*<0.002, Table 2). Ramakrishna *et al*. in their study among medical colleges in Orissa found that 34% students started tobacco use after joining medical college.[20]

One positive aspect came out of the study that 16.1% of users (Ever-exposed and current) medical respondents along with 7.9% of their nonmedical counterpart had given up tobacco use for more than 3 months. Although the difference in quitting rates was significantly high in the medical group (*Z*=2.02, *P*<0.0197, Table 2), but it was a welcome change in behavior, seen in both the groups, contrary to the popular belief that giving up tobacco is difficult. In a study (involving both the sexes of general people above 15 years of age), Jindal *et al*. showed a quitting rate of only 10% even after implementation of various antitobacco measures under tobacco control program.[21]

It was found that significantly higher proportion of nonmedical users was going on increasing their daily tobacco dose (*Z*=2.6, *P*<0.0094, Table 2).

On the other hand significantly higher number of medical users increased their daily use during mental tension (*Z*=2.89, *P*<0.0038, Table 2). This might be due to the tremendous academic pressure arising out of the vast medical course and career.

It was revealed that significantly higher proportion of medical students had correct knowledge about the uselessness of filter-tipped cigarette. It was also noteworthy that significantly higher number of medical participants was aware about different ill effects of tobacco use [Table 2].

About the pattern of tobacco use, the lion’s share was occupied by filter-tipped cigarette (65%), 9% by bidi (i.e., 65+9=74% were exclusively smokers), 7% by panparag-talab etc., 4% by pan-jarda-khaini (i.e., 11% were using exclusively smokeless tobacco), and more importantly 15% of subjects consumed all forms of tobacco.

About the modes/forms of use, it was found that proportion of smoking was significantly more in nonmedical group (*Z*=3.75, *P*<0.002) whereas both chewing and combination were significantly higher in medical students (Z=4.3, *P*<0.002, *Z*=2.7, *P*<0.007, Table 2). Kumari *et al*. in their study at Lucknow[17] found 87.5% smoker and 37.5% users of smokeless tobacco. Misbelieve about safety of filter-tipped cigarette and low awareness regarding health hazards of tobacco might be responsible for this significantly higher prevalence and proportion of smoking among the nonmedical respondents. On the other hand higher proportion of chewing and combining among medical category might be due to a more stringent smoking ban at hospital premises and less awareness about the danger of smokeless tobacco.

Welcome finding was that more than two-third of current users of both group had favorable attitude of quitting of this deadly habit [Table 2]. According to the report of GHPSS, 2006, M.H.F.W, GOI, there was over 71% current cigarette smokers and 73% current users of other tobacco products wanted to quit tobacco, while over 76% of current cigarette smokers tried to stop smoking cigarettes in the past year. Among ever smokers, 56.2% reported to have never received help/advice to stop smoking.[15] Some form of well-planned psychological counseling and empathic follow-up was just the need of the moment for this more than two-third of current users to curb this mortal habit and the rest one-third in favor of continuation of their present trend of use, our challenge, would need thoughtful planning and perseverance.

For 43–44% of users, the inspiration for tobacco adoption was reported to come out of curiosity (more in females, 60.0%) followed by peer pressure (25–30%). The males were influenced more by anxiety and film stars than females [Table 3]. This was contrary to the findings of Kumari *et al*. where tobacco initiation influenced by peer pressure reported to be on the top (78%) followed by curiosity.[17]

**Table 3:** Distribution of the users (both ever-exposed and current) by the nature of inspiration for initiation of tobacco use (*N*=276)

Influence/inspiration	Medical: No. (%)			Nonmedical: No. (%)			Both groups: No. (%)		
	Male	Female	Total	Male	Female	Total	Male	Female	Total
Curiosity	46 (43.81)	3 (42.86)	49 (43.75)	66 (44.0)	9 (64.3)	75 (45.7)	112 (43.92)	12 (57.1)	124 (44.92)
Peer pressure	32 (30.48)	1 (14.29)	33 (29.46)	36 (24.0)	3 (21.4)	39 (23.78)	68 (26.66)	4 (19.1)	72 (26.1)
To allay anxiety	19 (18.09)	−	19 (16.96)	21 (14.0)	−	21 (12.86)	40 (15.68)	−	40 (14.49)
Influenced by film stars	7 (6.67)	−	7 (6.25)	10 (6.67)	−	10 (6.1)	17 (6.66)	−	17 (6.2)
Others	1 (0.95)	3 (42.86)	4 (3.58)	17 (11.33)	2 (14.3)	19 (11.58)	18 (7.1)	5 (23.8)	23 (8.3)
Total	105 (100)	7 (100)	112 (100)	150 (100)	14 (100)	164 (100)	255 (100)	21 (100)	276 (100)

Regarding the dose or frequency of tobacco per day, it was revealed from the present study that on the whole more than half (56.32%) of current users consumed less than five times a day (proportion significantly more in nonmedical group, *Z*=2.39, *P*<0.0084), followed by 18.77% using 5–10 times (proportion significantly more in medical group), *Z*=2.37, *P*<0.0089), 17.95% using 11–20 times (almost equal in both groups) a day and about 7% (almost equal in both groups) current users were using more than 20 times a day. Almost all heavy users belonged to male category [Table 4].

**Table 4:** Frequency of daily tobacco use by the current users [N = 245 (n_1_=94 medical and n_2_=151 nonmedical)]

Frequency per day	Medical: No. (%)			Non-medical: No. (%)			*Z, P* values
	Male	Female	Total	Male	Female	Total	
<5	41 (46.07)	3 (60.0)	44 (46.8)	87 (62.6)	7 (58.33)	94 (62.3)	3.39, <0.002
5–10	24 (26.97)	1 (20.0)	25 (26.59)	17 (12.23)	4 (33.34)	21 (13.9)	3.37, <0.002
11–20	15 (16.85)	1 (20.0)	16 (17.02)	28 (20.14)	−	28 (18.5)	0.3, 0.7642
>20	9 (10.11)	−	9 (9.6)	7 (5.03)	1 (8.33)	8 (5.3)	1.21, 0.2262
Total	89 (100)	5 (100)	94 (100)	139 (100)	12 (100)	151 (100)	−

It was evident that tobacco use was to some extent less among medical respondents compared to their nonmedical peers; however, it should have been eliminated totally. In that sense, the observation by Kumari *et al*. in their study at Lucknow,[17] was partly correct that the medical knowledge was in vain to affect tobacco behavior of male medical students. The curbing would have been more if some thoughtfully planned, formal, routine anti-tobacco activities were templated and carried out in the concerned medical campuses. Poor enforcement of smoking ban, poor cessation help, and nonexistent formal cessation training in medical schools of India were noted (GHPSS).[15]

## CONCLUSION

Our “Anti-tobacco Acts” are mostly limited to papers and not being strictly enforced due to various reasons. People have minimum concern about their necessity, implications, and violate laws at random. Legislative actions and health-propagandas like posters, banners, leaflets, etc. can change tobacco behavior of people very little, as very few of them actually know the evils of tobacco in depth. Moreover, social acceptance of tobacco use, late and slow appearance of tobacco-related health hazards, mostly in chronic form, make people sometimes fail to correlate tobacco with morbidities caused by it and they remain less impressed by these statutory health warnings/danger symbols. Of particular interest in the developed countries has been the use of methods which address the social influences on tobacco use like parental smoking, peer pressure, stressful modern life, etc. We are to modify these psychosocial determinants of tobacco use to exterminate the tobacco menace.[14] Multipronged approach like strict enforcement of anti-tobacco laws, massive social mobilization for anti-tobacco movement using regular well planned anti-tobacco campaign, observation of “no tobacco/anti-tobacco” day, role play/drama/puppet show/folk song; demonstration, etc. can be beneficial as shown by different study results. Students may be benefitted by essay writing, focused/peer group discussion/debate, regular classes on tobacco impacts included in course curriculum starting from lower school level, cessation help/training etc. can be beneficial, as showed by different study results. Very rightly India has launched National Tobacco Control Program (NTCP) in its 11^th^ five-year plan, purposefully to chain the tobacco monster. We hope in the coming days all doctors will be real anti-tobacco teachers and leaders, and will help in curbing this public health menace by setting examples in front of community and guide people for quitting tobacco.
